# Phytochemical-Rich Colored Noodles Fortified with an Aqueous Extract of *Clitoria ternatea* Flowers

**DOI:** 10.3390/foods12081686

**Published:** 2023-04-18

**Authors:** Sy-Yu Shiau, Yanli Yu, Jing Li, Wenbo Huang, Haixia Feng

**Affiliations:** 1 Department of Food Nutrition and Safety, Sanda University, Shanghai 201209, China; 2 Department of Food Science and Technology, Tajen University, Pingtung County 90741, Taiwan

**Keywords:** natural pigment, butterfly pea, anthocyanin, antioxidant, noodle, color

## Abstract

*Clitoria ternatea* (CT) flowers are rich in phytochemicals. An innovative approach was taken to utilize CT flower extract (CTFE) as a functional ingredient with natural pigment by incorporating it into noodles. The aim of this study was to examine the effect of the CTFE amount (0–30%) on the color, texture, phytochemicals, and sensory quality of both dried and cooked noodles. Dried noodles with 30% CTFE had the highest total anthocyanins (9.48 μg/g), polyphenols (612 μg/g), DPPH radical scavenging capacity (165 μg TE/g), and reducing power (2203 μg TE/g). Cooking resulted in a significant decrease in the anthocyanin levels and blue color, while also increasing the greenness of the noodle. Both dried and cooked noodles with 20–30% CTFE showed a significantly higher color preference compared to the control sample. Despite a significant reduction in the cutting force, tensile strength, and extensibility of cooked noodles with 20–30% CTFE, the sensory attributes such as flavor, texture, and overall preferences were similar to those of noodles with 0–30% CTFE. Blue noodles with high phytochemicals, antioxidant activities, and desirable sensory qualities can be produced by the incorporation of 20–30% CTFE.

## 1. Introduction

Noodle products are a pleasant staple food in Asia. Noodle quality, such as color, texture, and nutrition, is affected by the ingredients and processing methods [1,2]. Since humans continuously seek a healthy, long life, and colorful food products with good quality, there is a growing worldwide trend toward the manufacturing of food products with colored functional ingredients such as grape pomace [3], black rice bran [4], and pitaya peel [5].

The perennial leguminous herb *Clitoria ternatea* (CT) (or butterfly pea) is widely cultivated in tropical regions, and its flower can be utilized in the food industry as a natural pigment [6]. Since CT flowers and extracts are rich in bioactive substances, they have antioxidation, anti-inflammation, antibacterial, antidiabetic, and antitumor activities [7,8,9]

Anthocyanin is a water-soluble natural pigment that mainly occurs in the flowers, leaves, fruits, and seeds of plants. The structure of anthocyanins consists of an anthocyanidin (aglycon), sugars, and/or organic acids [7,10]. The main anthocyanidins that occur in plants are pelargonidin, cyanidin, peonidin, delphinidin, petunidin, and malvidin. The CT flower is rich in blue polyacylated anthocyanins, which are known as ternatins and are derivatives of delphinidin 3, 3′, 5′-triglucoside [6,11]. Currently, researchers have identified about eighteen delphinidin and cyanidin derivatives from CT flower [9,12,13]. Anthocyanins can be used in intelligent films to monitor the freshness of food [14] and offer many health-promoting benefits such as anticancer, antidiabetic, and vascular protective effects [15].

The stability of anthocyanins relates to the chemical structure, temperature, pH, light, oxygen, and water activity [6,16,17]. Voss et al. [18] indicated that 10-catechyl-pyranoanthocyanins, formed by reacting anthocyanins with caffeic acid, displayed an excellent thermal stability. Anthocyanins were generally more stable at a lower temperature, pH, and water activity without oxygen and light. The extract of CT anthocyanin was very stable at pH 4.0–8.0, and with an increase in the pH from 0.5 to 13, the color of the extract changed from purple, red, and blue to green and yellow [19]. Anthocyanins extracted from CT flowers demonstrated stability at temperatures ranging from 60 to 70 °C within a pH range of 3.6 to 5.4. However, it was observed that the rate of thermal degradation raised significantly beyond 70 °C [6].

Some articles concerning the effects of anthocyanins on noodle and pasta properties have been reported. Pasta quality including antioxidant activity, anthocyanin, and fiber contents could be significatly improved by purple wheat (bran) and black rice bran [4,20,21]. An addition of up to 5% of extracts from the coat of black soybean could enhance the nutritional values of noodles with a satisfactory textural quality [2]. CT flowers are rich in blue anthocyanins and other bioactive compounds. Therefore, incorporating the flowers or their extracts into noodle products has the potential to enhance the color, nutritional value, and antioxidant activity of the final product. Yet, reports on the physicochemical properties of CT flower-containing noodles and on the stability of anthocyanins during the cooking process of noodles and pasta are rarely few. The aims of this study were as follows: (1) to investigate the color, texture, phytochemicals, antioxidative, and sensory properties of both dried and cooked noodles fortified with various CTFE amounts, and (2) to investigate the thermal stability of phytochemicals during the cooking process of noodles.

## 2. Materials and Methods

### 2.1. Materials

Wheat flour with 11.00% crude protein and medium gluten strength was purchased from Taichung Chia-Fha Enterprise Co. (Taichung, Taiwan) Fresh flowers of *Clitoria ternatea* (CT) were bought from a local market. All the chemicals and reagents used were of analytical grade.

### 2.2. Preparation of CTFE

Dry CT sample was obtained by drying (50 °C for 12 h) fresh flowers. Factors affecting the extraction yield of anthocyanins from CT flowers were solvent type, the ratio of substrate and solvent, extraction temperature, and time. Water heated to between 40 and 100 °C has been effectively used to extract anthocyanins from the flowers of butterfly pea [6], due to the economy, convenience, and non-toxicity. In the preparatory study for extracting anthocyanin from dried CT flowers, the total anthocyanin content of the aqueous extracts gradually increased from 4.79 to 24.55 mg/L when the extraction temperature increased from 30 to 90 °C, while maintaining a fixed extraction time of 30 min. To prepare aqueous extracts, 6 g of dried CT flowers were mixed with 300 mL of distilled water (90 °C) in this study. The mixture was then stirred on a hot plate with a magnetic stirrer for 30 min at 90 °C to extract bioactive compounds. After cooling, the extract was passed through a filter using a Buchner funnel and vacuum suction. Then, the filtered extract was mixed with distilled water until the final volume reached 300 mL.

### 2.3. Noodle Preparation

Wheat doughs with a 35.00% moisture content (wet basis) were produced according to the method [5]. Basic wheat dough was prepared by mixing 500 g of wheat flour (11.97% moisture) and distilled water (177.2 g). For making the dough fortified with 10%, 20%, and 30% CTPE, the amounts of CTFE and distilled water added were 50 and 127.9 g, 100 and 78.6 g, as well as 150 and 29.3 g, respectively.

After being allowed to rest at room temperature (approximately 26 °C) for 10 min, the dough was kneaded by hand to develop a cohesive mass. Next, the noodle dough was rolled into a sheet using a dough sheeter (Model HC1-A, Dean Food Equipment Co., Kaohsiung, Taiwan). The dough sheet underwent a gradual reduction in thickness by passing through roll gaps of 9.0, 7.0, 5.5, 4.2, 2.9, 1.5, and 0.8 mm until it reached a final thickness of approximately 1.6 mm. Subsequently, the dough sheet was sliced into raw noodles using a noodle cutter with a width of 3 mm. The noodle cutter used for this process was the DRB-36 model, manufactured by the Dean Food Service Equipment Co., Ltd. Dried noodles with about 11.5% of moisture content were prepared by air drying overnight in an air-conditioned room (26 °C, 60% RH).

To prepare cooked noodle samples, around 30 g of the dried noodles were boiled with 400 mL of water until the white core was no longer visible. After cooking, the noodles were transferred to a container filled with cold water at a temperature of 25 °C and left to soak for 20 s. Once cooled, the cooked noodles were promptly placed into sealable plastic bags and analyzed as soon as possible. Dry and cooked noodles with CTFE concentrations ranging from 0 to 30% were prepared by three replications for analysis of their physicochemical properties.

### 2.4. Total Anthocyanin Content (TAC)

TACs of CTFEs and noodles were determined by pH differential methods [22,23]. Firstly, both dried and cooked noodle samples (2 g) were extracted with 10 mL of 80% ethanol for 30 min, and then were centrifuged for 10 min at 6000× *g*. To determine TAC, the supernatant was collected and the extraction process was repeated. The combined supernatant and CTFE were analyzed using spectrophotometry. The extract sample (1 mL) was mixed thoroughly with 4 mL of pH 1.0 buffer containing 0.025 M KCl (adjusted by HCl), and another 4 mL of pH 4.5 buffer of 0.4 M sodium acetate. The resulting solutions were then measured for absorbance at 520 and 700 nm. The TAC values of both the CTFE and noodles were calculated using the following equation:

TAC (μg/g, DB) = (A × F × MW × 1000 × V)/(ε × *x* × W) (1)

where A = [(A_520nm_ − A_700nm_)*_pH 1.0_* − (A_520nm_ − A_700nm_)*_pH_*_4.5_]; F: dilution factor; MW: 449.2 g/mol of cyanidin-3-glucoside; V: volume of extract (mL); ε: 26,900 L/mol.cm, molar extinction coefficient of cyanidin-3-glucoside; x: path length of cuvette (cm); W: sample weight (g); and DB: dry weight basis.

### 2.5. Free Polyphenolic Content (FPC)

The method specified in reference [5] was employed to repeatedly extract the free form of total polyphenols from the noodles using 80% ethanol. The combined supernatant obtained from the extraction was used to determine the FPC and antioxidant activities. For FPC determination, the Folin–Ciocalteau method, as described in reference [24], was utilized. The absorbance was measured at 725 nm, and a standard curve was generated using ferulic acid. The FPC value was expressed as μg of ferulic acid equivalent (FAE) per gram of noodle (on a dry basis).

### 2.6. Antioxidant Activity

The radical scavenging activity of dried and cooked noodles’ ethanolic extracts was assessed using the DPPH method, with Trolox as the standard. The spectrophotometric analysis was carried out at 517 nm [25], and the results were expressed in terms of Trolox equivalent (TE) per gram of noodles on a dry basis (DB). Furthermore, the reducing power of the ethanolic extract was determined using the ferric chloride and potassium ferricyanide method [26], and the development of a ferric–ferrous complex was observed by spectrophotometry at 700 nm. The results were also expressed in terms of TE per gram of noodles (DB).

### 2.7. Color of Noodle

The color of dried and cooked noodles was measured using a ColorFlex colorimeter from Hunter Associates Laboratory, Inc., located in Reston, VA, USA. Each noodle sample underwent six color measurements. The L* value, ranging from 0 to 100, indicates the level of whiteness or blackness. The a* and b* values indicate the degree of redness and yellowness or greenness and blueness, respectively, with positive values indicating the former and negative values indicating the latter. To calculate the white index (WI), the following equation was used:

(2) $$WI=100-\sqrt{{\left(100-L^{*}\right)}^{2}+a^{*2}+b^{*2}}$$

### 2.8. Breaking and Cutting Forces of Noodle

Both breaking and cutting forces of the noodle samples were measured using the method [5]. To determine the breaking force (N), a single dried noodle measuring 8 cm in length was placed horizontally on the platform of a three-point bend rig (HDP/3PB) attached to a texture analyzer (TA-XT2i, Stable Micro Systems, Godalming, UK). The test was conducted at a speed of 1.0 mm/s, and the maximum force on the compression curve was recorded as the breaking force (mN). Cutting force of five cooked noodle strands was determined using a knife probe (A/LKB-F). Test speed was set to 0.5 mm/s and compression depth was set to 1.0 mm. The cutting force (mN) of the cooked noodles was read from the maximum force on compression curve. The measurements of both breaking and cutting forces of noodle samples were conducted in six repetitions for each treatment.

### 2.9. Tensile Strength and Extensibility of Noodle

Tensile properties of one cooked noodle were determined by the texture analyzer operated in tension mode and equipped with a spaghetti/noodle tensile rig. Both pretest and test speeds were set to 2.0 mm/s. Tensile strength (TS) and extensibility (E) of the cooked noodle can be measured by reading the maximum force and the distance at extension limit points during a tensile test. Tensile measurement was performed in 6 replications for each treatment.

### 2.10. Stress Relaxation of Noodle

According to the method of Pan et al. [27], three repetitions of stress relaxation testing were performed on cooked noodles for each treatment using the texture analyzer rigged with a P20 probe. Over a period of 240 s, the force remaining in the sample during testing at a 20% strain was recorded continuously as a function of time. Percent stress relaxation (%SR), a simple parameter for food viscoelasticity, can be obtained from the following equation [28]:

(3) $$\%SR=\frac{F_{0}-F_{t=20}}{F_{0}}\times 100$$

where F_0_ is initial force, F_t=20_ is the force at 20 s after the initial strain was achieved. Furthermore, the data of stress relaxation were non-linearly regressed by using Peleg–Normand model [29], Equation (4).

(4) $$\frac{F_{0}t}{F_{0}-F(t)}=k_{1}+k_{2}t$$

where F_0_ is initial force, F(t) is the momentary force at time t, and both k_1_ and k_2_ values are constants.

### 2.11. Cooking Quality of Noodle

Cooking quality of noodle was executed according to the method [5]. As mentioned earlier, the dried noodle samples were cooked until the white core was no longer visible, and the appropriate cooking time was determined to be 10 min. The weights of the dried and cooked noodles, as well as the solids in the cooking water, were measured. The moisture content and cooking loss of cooked noodles could be determined using the gravimetric method, with three replications conducted for each treatment of noodle sample.

### 2.12. Sensory Evaluation

For consumers’ sensory evaluation of the noodles enriched with various CTFE levels, thirty untrained panelists (16 females and 14 males, within the age range of 19 to 60 years) were invited by students and employees at the university. Each panelist was given four noodle samples to assess at the same time. Each panelist was served four samples of dried or cooked noodles, with each sample weighing approximately 30 or 60 g and placed on a white plate labeled with a three-digit code. The samples were presented to the panelists simultaneously. They were then asked to rate the noodles using a 7-point hedonic scale based on color, flavor, texture, and overall acceptability. The highest value represents the highest degree of acceptance (7 = extremely like, 1 = extremely dislike).

### 2.13. Statistical Analysis

The data from triplicate samples of the same type of noodles but different treatments were analyzed using SPSS software version 20.0 (SPSS, Chicago, IL, USA). To ascertain the statistical significance (*p* < 0.05) of the variations observed among the values, one-way ANOVA and Duncan’s new multiple range tests were used.

## 3. Results and Discussion

### 3.1. Total Anthocyanins of Noodle

**Figure 1:**
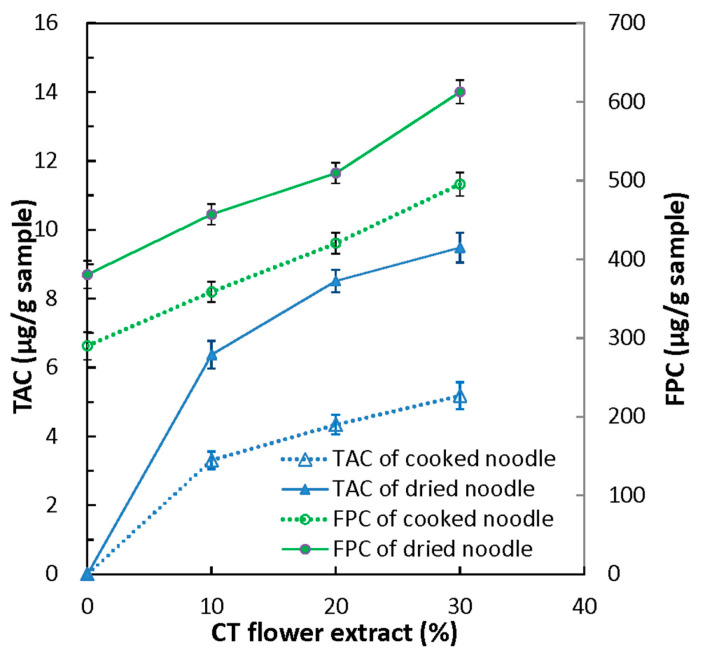
Total anthocyanin content (TAC) and free phenolic content (FPC) of dried and cooked noodles with various CTFE levels.

The TAC of the CTFE prepared in this study was 24.55 mg/L or 2668 mg/kg (DB). It was similar to the study [30] in that the extracts of the CT flower via an ultrasound-assisted extraction with water for 30–60 min at 40–80 °C contained 1050–2190 mg cyanidin-3-glucoside/kg (DW). The TACs of dried and cooked noodles were highly augmented by the levels (0–30%) of the CTFE (Figure 1), which ranged from 0 to 6.37–9.48 μg/g noodle (DB) for the dried noodle and ranged from 0 to 3.31–5.18 μg/g noodle (DB) for the cooked form, respectively. Thus, the TAC of dried noodles was reduced by about 45.34–49.00% during the cooking process. The decrease in the TAC of the noodle may be attributed to thermal degradation [6,31] and leaching of anthocyanins, as evidenced by the mild blue color of the cooking water when the noodle fortified with CTFE was cooked for 10 min. The use of black soybean coat, black rice bran, and purple wheat could have similar enriching effects on the anthocyanin content of both noodles and pasta [2,4,20].

Although the thermal degradation mechanisms of anthocyanins are not yet fully understood, it is known that their chemical structure plays a crucial role in their stability. Research has shown that polyacylated and polyglycosylated anthocyanins are more resistant to changes in heat and pH [31]. For example, tenatins, which are the main polyacylated anthocyanins found in CT flowers, are relatively stable under acidic and neutral conditions [11]. However, anthocyanins can undergo degradation during heat treatment as a result of deacylation, deglycosylation, oxidation, and the cleavage of covalent bonds [6,19]. This study also found evidence of thermal degradation of anthocyanins, as the dried CTFE-fortified noodle had a higher TAC than the cooked noodle.

In a study by Eliasova et al. [16], it was found that 45–51% of anthocyanins were lost during the baking stage of blue-grain wheat bread. Similarly, only 41.8% of the initial anthocyanin content was retained when cupcakes enriched with anthocyanin extract from CT flowers were baked at 170 °C for 20 min [32]. Additionally, the total anthocyanin content (TAC) of steamed bread with CT flower extract was reduced by approximately 36–40% after steaming [23]. These findings suggest that the stability of anthocyanins in food systems is influenced by factors such as the intensity, duration, and pattern of heating during thermal processing. However, there are limited reports on the thermal degradation of anthocyanins in noodle and pasta products, making it difficult to compare the results of this study with the other related literature on wheat-based products.

### 3.2. FPC and Antioxidant Capacity of Noodle

CT flowers contained a high level of phytochemicals (such as anthocyanins, phenolic acids, and flavanols), which enhanced the antioxidant activity of enriched foods [6,33]. The CTFE used in this study had 65,401 μg FAE/g for the FPC, 15,080 μg TE/g for the DPPH scavenging activity, and 168,476 μg TE/g (DB) for the reducing power.

**Figure 2:**
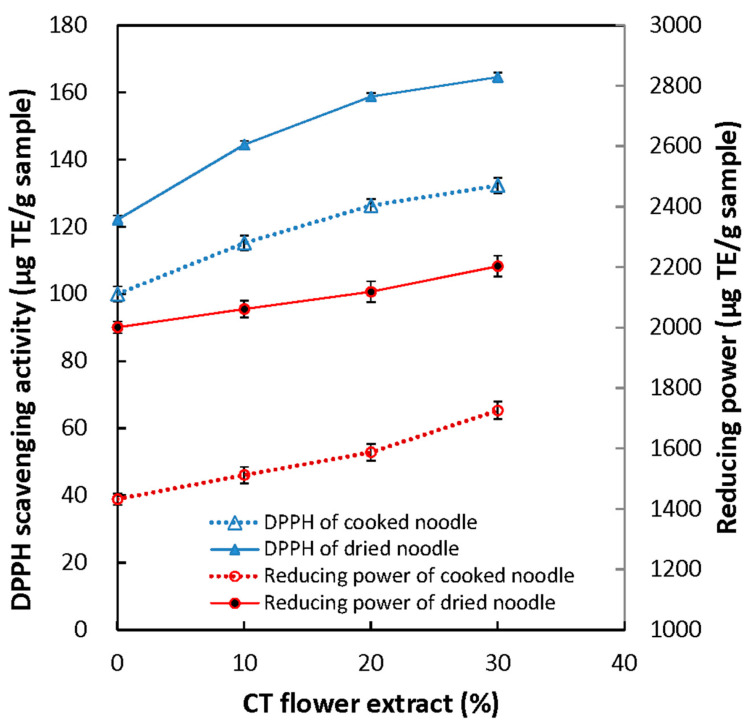
DPPH scavenging capacity and reducing power of both dried and cooked noodles fortified with various CTFE amounts.

The results in Figure 1 and Figure 2 showed that the FPC and antioxidant activities of both dried and cooked noodles obviously enhanced by increasing the amount of CTFE. Increasing the amount of CTFE in the dried noodles from 0 to 30% resulted in significant improvements in their FPC, DPPH radical scavenging capacity, and reducing power. The FPC increased by 20.1–61.0%, the DPPH radical scavenging capacity by 18.3–34.8%, and the reducing power by 3.0–10.1%. For the cooked noodles, FPC and two antioxidant activities improved by 23.6–70.8%, 15.2–32.3, and 5.6–20.5%, respectively. Moreover, phytochemicals (TAC and FPC) of the noodles positively correlated with the antioxidant activities (DPPH radical scavenging capacity and reducing power) (r > 0.87, *p* < 0.05). The findings of this study are consistent with previous research demonstrating that bread [34], sponge cake [35], steamed bread [23], and breakfast cereals [33] fortified with CT flower extract had significantly higher levels of total polyphenols and antioxidant capacity compared to the control products.

### 3.3. Color of Noodle

**Figure 3:**
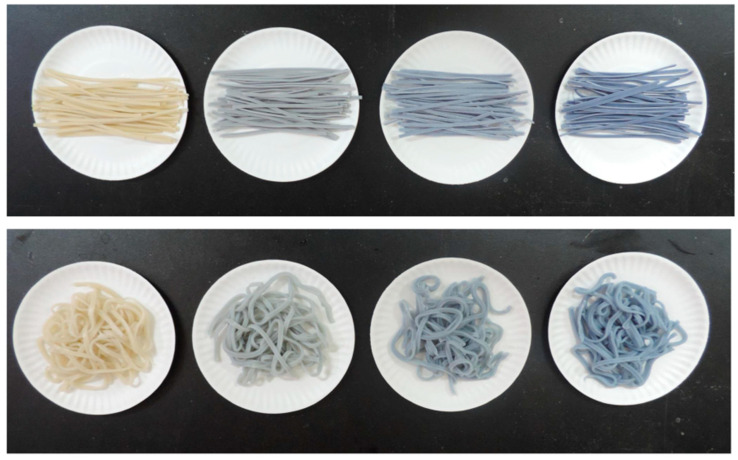
Appearances of dried noodle (**upper**) and cooked noodle (**lower**) fortified with 0, 10, 20, and 30% CTFE, respectively (from **left** to **right**).

Food color is one of the important attributes in evaluating food quality. Due to the rich ternatins, the petals of CT and CTFE appeared navy blue. The control noodle displayed a white appearance in Figure 3, whereas the dried and cooked noodles containing 10–30% CTFE exhibited color variations ranging from light blue to blue. The effects of the CTFE levels (0–30%) on the color parameters of dried and cooked noodles are listed in Table 1. As the CTPE level increased, both Hunter L* and WI values of dried and cooked noodles significantly decreased, but the greenness (-a*) and blueness (-b*) of the noodles appeared to be remarkably increased. After the cooking process, the cooked CTFE-fortified noodle had an obviously higher brightness, WI, and greenness than the uncooked noodle. It was observed that the cooked noodles fortified with CTFE had lower blueness levels compared to their dried counterparts, which was consistent with the decrease in TAC shown in Figure 1. This could be attributed to the thermal degradation of ternatins from their blue neutral quinonoid base to the yellow chalcone form [19], as well as the release of anthocyanins into the cooking water of the noodle. Marpaung et al. [36] indicated that the color degradation of CT anthocyanins at pH 7 occurred through the deacylation, while at pH 4, it occurred through the unfolding of the hydrophobic interaction.

During processing, the crumb and crust of the sponge cake containing 5–20% CT dried extract exhibited a greenish and brownish color, which can be attributed to the pH change [35]. Additionally, the bread crumb of anthocyanin-rich bread containing 30% blue-seeded maize flour changed color to a range of red shades [37]. The addition of citrus juice to CT ice cream led to color changes from blue to purple as its pH value decreased from 6 to 4 [38]. In this study, both dried and cooked noodles enriched with CTFE retained their blue color even after undergoing various processing stages such as mixing, sheeting, drying, and cooking. Previous research by Gamage et al. [6] highlighted the potential of blue anthocyanins from CT flowers to serve as a natural blue food colorant in neutral and mildly acidic food products. These observations suggest that factors such as the heating method, pH, and chemical structure of anthocyanins are crucial in determining the color stability of foods that are fortified with anthocyanin-rich ingredients.

### 3.4. Textural, Rheological and Cooking Properties of Noodle

The texture and viscoelasticity of noodle products are chiefly influenced by the integrity of the gluten network. Table 2 lists the effect of various CTFE levels on the texture and cooking properties of noodles. Although the breaking force of dried noodles was not significantly affected by the CTFE level, cooked noodles fortified with high CTFE level (20% or 30%) had a significantly lower cutting force and tensile properties than the control noodles. Hence, the addition of a high CTFE level might result in a weaker and less extensible cooked noodle, due to the dilution of the gluten protein and protein–anthocyanin interaction [11,39,40].

The hardness, elasticity, and cohesiveness of cooked noodles with 2.5–5% of vacuum-dried anthocyanins-rich extract from black soybean coat were not significantly changed (*p* > 0.05) [2]. However, cooked noodles fortified with higher levels of extract (7.5% or 10%) showed a significant decrease in the hardness, elasticity, and cohesiveness, and the continuous network structure of gluten was destroyed at the higher extract levels [2]. The incorporation of anthocyanin-rich black rice bran to the pasta led to a shorter cooking time, pasta firmness, and stickiness with higher cooking losses, since the bran containing high fiber disrupted the protein–starch matrix [4]. Boiled pasta supplemented with purple sweet potato powder (2.5–10%) had a lower hardness, but its elasticity was insignificantly (*p* > 0.05) influenced by the powder amount [41].

Food viscoelasticity can be determined by the stress relaxation experiment. The stress relaxation data of cooked noodles can be fitted well by the Peleg–Normand model (Equation (4)), with an R^2^ > 0.999. Cooked noodles with 0–30% CTFE had similar %SR (53.44–54.39), k_1_ (10.02–10.79 s), and k_2_ (1.499–1.506) values. This indicated that the elasticity of cooked noodles was not influenced by the CTFE level. In Table 2, different CTFE levels do not significantly affect the moisture content of the cooked noodle (64.39–65.58%) and the cooking loss (4.28–4.43%), which is low and acceptable.

### 3.5. Sensory Evaluation of Noodle

The quality of the cooked noodles determined by different analytical instruments in Table 1 and Table 2 was significantly or insignificantly affected by various CTFE levels. An objective instrumental measurement generally has a better sensitivity than a subjective sensory evaluation. The sensory scores of consumers for dried and cooked noodles fortified with different levels of CTFE are presented in Table 3. The addition of various levels of CTFE did not have a significant impact on the flavor acceptability of dried noodles (*p* > 0.05). However, dried noodles fortified with 20–30% CTFE had a significantly higher color acceptability compared to the control sample. Similarly, cooked noodles fortified with 20–30% CTFE also had a significantly higher color acceptability than the control sample. In terms of the flavor, texture, and overall acceptability, there were no significant differences (*p* > 0.05) observed in the cooked noodles fortified with 0–30% CTFE. These findings suggest that the addition of 20–30% CTFE can be a viable option to produce anthocyanin-rich colorful noodles, which can help increase the phytochemical intake without compromising the sensory acceptability. Santiago et al. [41] found that pasta supplemented with 7.5% and 10% of purple sweet potato powder had a similar sensory quality to the control pasta. No differences were found in the overall acceptability between sponge cake with 5–20% of CT extract and the control [35]. However, cupcakes fortified with the anthocyanin extract of the CT flower exhibited a higher color, flavor, and overall acceptability when compared to the control sample [32].

## 4. Conclusions

Due to their rich blue pigments and bioactive compounds, *Clitoria ternatea* (CT) flowers have the potential to be used as a natural colorant and functional ingredient in noodle products. The CTFE level significantly affected the color, antioxidant activity, texture, and sensory attributes of both dried and cooked noodles. An addition of 10–30% CTFE resulted in a significant improvement in the TAC, FPC, radical DPPH scavenging capacity, and reducing power of the noodles, which exhibited a light blue–blue coloration. However, during the cooking process, there was a noticeable reduction in the TAC and blueness of the noodle, along with an increase in the greenness, due to the thermal degradation and leaching of anthocyanins. Despite the significant reduction in the cutting force and tensile properties of the cooked noodle with 20–30% CTFE, the CTFE levels did not significantly affect the sensory flavor, texture, and overall acceptability of the noodle. This study demonstrates that blue noodles containing high bioactive compounds and antioxidant capacities with acceptable sensory qualities can be produced by the incorporation of 20–30% CTFE.

## Figures and Tables

**Table 1:** The color parameters of noodles fortified with different CTFE levels.

CTFE (%)	Kind	L*	a*	b*	WI
0	Dried noodle	70.72 ± 0.74 ^b^	1.24 ± 0.22 ^a^	17.69 ± 0.56 ^a^	65.77 ± 0.90 ^b^
10	Dried noodle	58.40 ± 0.40 ^e^	−3.35 ± 0.18 ^c^	−1.30 ± 0.25 ^d^	58.25 ± 0.42 ^e^
20	Dried noodle	54.97 ± 0.95 ^g^	−3.81 ± 0.23 ^d^	−7.11 ± 0.22 ^f^	54.25 ± 0.96 ^g^
30	Dried noodle	46.73 ± 0.59 ^h^	−3.95 ± 0.18 ^d^	−11.00 ± 0.19 ^h^	45.46 ± 0.59 ^h^
0	Cooked noodle	73.70 ± 0.41 ^a^	−1.54 ± 0.14 ^b^	8.04 ± 0.25 ^b^	72.46 ± 0.36 ^a^
10	Cooked noodle	64.50 ± 0.67 ^c^	−4.84 ± 0.27 ^e^	−0.41 ± 0.18 ^c^	64.17 ± 0.67 ^c^
20	Cooked noodle	60.38 ± 0.60 ^d^	−5.95 ± 0.19 ^f^	−6.42 ± 0.17 ^e^	59.42 ± 0.60 ^d^
30	Cooked noodle	56.85 ± 0.31 ^f^	−6.67 ± 0.13 ^g^	−9.20 ± 0.12 ^g^	55.38 ± 0.32 ^f^

The reported values represent means ± standard deviations (*n* = 6), and any means within the same column that have different letters are considered to be significantly different (*p* < 0.05). CTFE: *Clitoria ternatea* flower extract; WI: white index.

**Table 2:** Influence of CTFE amount on texture and cooking properties of noodles.

CTFE (%)	Breaking Force (mN)	Cutting Force (mN)	Tensile Strength (mN)	Extensibility (mm)	Moisture Content (%)	Cooking Loss (%)
0	1779 ± 85 ^a^	1732 ± 67 ^a^	399.7 ± 24.5 ^a^	55.21 ± 3.07 ^a^	64.77 ± 0.47 ^a^	4.31 ± 0.13 ^a^
10	1760 ± 98 ^a^	1687 ± 66 ^ab^	393.3 ± 10.7 ^a^	52.49 ± 2.68 ^a^	64.39 ± 0.59 ^a^	4.43 ± 0.11 ^a^
20	1674 ± 82 ^a^	1595 ± 70 ^bc^	385.8 ± 16.6 ^a^	46.41 ± 3.02 ^b^	64.93 ± 0.51 ^a^	4.28 ± 0.12 ^a^
30	1712 ± 73 ^a^	1550 ± 57 ^c^	323.8 ± 10.1 ^b^	39.90 ± 2.67 ^c^	65.58 ± 0.54 ^a^	4.42 ± 0.15 ^a^

The reported values represent means ± standard deviations (n = 3), and any means within the same column that have different letters are considered to be significantly different (*p* < 0.05).

**Table 3:** Sensory evaluation of dried or cooked noodle fortified with various CTFE levels.

	Dried Noodle		Cooked Noodle			
CTFE (%)	Color	Flavor	Color	Flavor	Texture	Overall
0	4.45 ± 1.26 ^b^	4.46 ± 1.04 ^a^	4.31 ± 1.13 ^b^	4.26 ± 1.09 ^a^	5.17 ± 1.03 ^a^	5.21 ± 1.02 ^a^
10	4.35 ± 1.17 ^b^	4.50 ± 1.38 ^a^	4.37 ± 1.26 ^b^	4.43 ± 0.95 ^a^	4.92 ± 1.29 ^a^	4.93 ± 1.16 ^a^
20	5.56 ± 1.35 ^a^	4.43 ± 0.87 ^a^	5.25 ± 1.30 ^a^	4.96 ± 1.12 ^a^	5.16 ± 1.45 ^a^	5.25 ± 1.23 ^a^
30	5.93 ± 0.92 ^a^	4.70 ± 0.92 ^a^	5.72 ± 1.05 ^a^	4.83 ± 1.36 ^a^	5.03 ± 1.67 ^a^	5.36 ± 1.15 ^a^

The reported values represent means ± standard deviations (n = 30), and any means within the same column that have different letters are considered to be significantly different (*p* <0.05).

## Data Availability

Data are contained within the article.
